# *Pyrocystis noctiluca* represents an excellent bioassay for shear forces induced in ground-based microgravity simulators (clinostat and random positioning machine)

**DOI:** 10.1038/s41526-017-0016-x

**Published:** 2017-04-24

**Authors:** Jens Hauslage, Volkan Cevik, Ruth Hemmersbach

**Affiliations:** DLR (German Aerospace Center), Institute of Aerospace Medicine, Gravitational Biology, Linder Höhe, Cologne, 51147 Germany

## Abstract

Ground-based facilities, such as clinostats and random positioning machines aiming at simulating microgravity conditions, are tools to prepare space experiments and identify gravity-related signaling pathways. A prerequisite is that the facilities are operated in an appropriate manner and potentially induced non-gravitational effects, such as shearing forces, have to be taken into account. Dinoflagellates, here *P. noctiluca*, as fast and sensitive reporter system for shear stress and hydrodynamic gradients, were exposed on a clinostat (constant rotation around one axis, 60 rpm) or in a random positioning machine, that means rotating around two axes, whose velocity and direction were chosen at random. Deformation of the cell membrane of *P. noctiluca* due to shear stress results in a detectable bioluminescence emission. Our results show that the amount of mechanical stress is higher on an random positioning machine than during constant clinorotation, as revealed by the differences in photon counts. We conclude that one axis clinorotation induced negligible non-gravitational effects in the form of shear forces in contrast to random operation modes tested. For the first time, we clearly visualized the device-dependent occurrence of shear forces by means of a bioassay, which have to be considered during the definition of an appropriate simulation approach and to avoid misinterpretation of results.

## Microgravity simulators: Bio-reporters illuminate false signals

Earth-based laboratories can now assess the accuracy of tools used to simulate living organism growth and behaviour in space with bioluminescent assays. Researchers often use rotating machines to minimize gravity effects during the design of extra-terrestrial experiments with plants, cells, and small animals. Jens Hauslage from the DLR German Aerospace Center and colleagues report that device-specific shear forces produced during mechanical movements may cause misinterpretations of initial test data. They developed a biosensor based on marine plankton, known as dinoflagellates, which have cell membranes that naturally emit light when touched by predators. Calibrating this bioluminescence against mechanical stress helped determine the top-like, 2D rotations of ‘‘clinostat’’ devices provided microgravity-like conditions. However, the unexpected 3D movements of Random Positioning Machines generated enough shear force to impact studies of cell signaling pathways or metabolic reactions.

## Introduction

Experiments in space need preparation and a solid base of ground control experiments, which will later help to identify and explain microgravity-induced effects. To generate microgravity analog conditions for biological organisms in an Earth-based laboratory, different methods have been developed and are in use by scientists. Clinostats and random positioning machines (RPM) are the common facilities to treat cell cultures, small animals and plants aiming to neutralize the effect of gravity.^1–3^

**Fig. 1:**
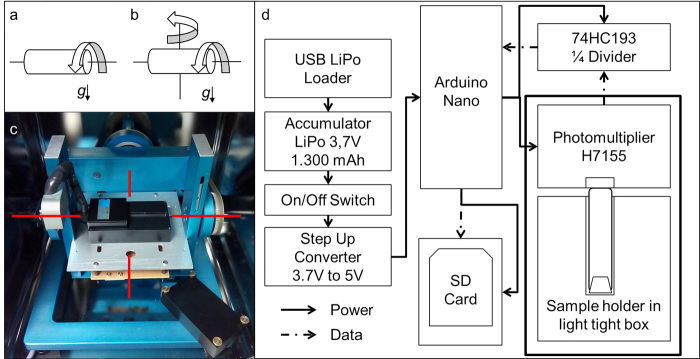
**a** Clinostat mode. The sample cuvette was rotated in one axis. **b** RPM mode. During the RPM mode, the second axis in gimbal mount was additionally rotated. **c** Desktop RPM from Dutch Space integrated in a temperature controlled, dark incubator. On the top of the experiment platform the light tight PMT box is visible (*opened*). Beneath the experiment platform, the circuit board with the Arduino, SD card, power supply and regulators are attached. The *red lines* indicate the rotation axes. The inner frame of the RPM was used as clinorotation axis (*horizontal lines*). **d** Schematic of the functional set-up. An USB loader unit will recharge the lithium polymer accumulator. The voltage from the accumulator is converted to 5 V via a step up converter driving the Arduino. The PMT is powered over the Arduino and is sending the digital counts over a 74HC193 divider to the input pin 5 of the Arduino. The Arduino writes the counted values and record time to a comma separated value file on the SD card over SPI bus

Unfortunately, experiment description often lack detailed reports of the hardware that contain the test systems, of the operational procedures, such as the method and cycle of fluid (medium) exchange as well as a critical discussion of non-gravitational effects achieved by the physical principle applied. The principle of a two-dimensional (2D) clinostat is the following: samples in containers are rotated around one axis, which is positioned perpendicular to the direction of the gravity vector (Fig. 1a). Under optimal conditions, the diameter of the containers is kept small (in the range of a few mm) and the objects are placed in the center of rotation in order to keep accelerations as minimal as possible.^2, 4^ Zero head space and complete filling of the sample containers are essential to decrease mechanical disturbances. A 2D clinostat is constantly operated in one direction thereby inducing a static change of the gravity vector in relation to the rotated sample. In turn, sedimentation is prevented and small bodies will describe floating circles in the media comparable to the floating conditions under real microgravity. The diameter of the circles depends on the speed of rotation; the faster the rotation, the smaller the circles, while too fast rotation results in centrifugal acceleration.^1^

Three-dimensional (3D) clinostats and RPMs are also experimental platforms for ground-based experiments. Both are based on the same principle having two rotation axes in a gimbal mount (Fig. 1b, c). An algorithm controls the motors with respect to moving, accelerating, or changing the direction.^5–7^ The mode of operation is commonly used for the terminology: while 3D clinostats are continuously rotating by changing the rate of rotation of the two motors at random,^5^ a RPM is characterized by not only changing the velocity but additionally the direction of rotation in a real random mode.^2, 3^

Equipment of these devices with optical applications already revealed differences in the movement of particles and cells.^1^ It is assumed that induction of mechanical stress due to the operation mode of the ground-based facility cannot be excluded.

Here, we present a validation approach to demonstrate device-specific-induced shear forces and in turn stimulation of a biosensor. Dinoflagellates have been shown to be model organisms to visualize shear and hydrodynamic forces in fluids with one of the fastest mechanosensitive reporter systems. The delay time between stimulus and bioluminescent response is in the range between 15 to 20 ms (refs 8–10).

Mechanical shearing of dinoflagellates induces intracellular signaling, which is only partly understood. A cellular shear receptor has been postulated in this cascade, which triggers the increase in cytosolic Ca^2+^, in turn the reaction between luciferin and luciferase and thereby the emission of light.^11–13^

The ecological reason for bioluminescence is defense and hunting. Predators touch the prey when feeding on them and produce water streams due to their swimming activities that in turn results in luminescent dinoflagellates, which might disturb the predator´s activity.^14^ Dinoflagellates are prey of especially small crustaceans. The further biological advantage of bioluminescence is obviously the attraction of secondary predators.^15, 16^ It was demonstrated that both fish^15^ and a cephalopod (Sepia)^17^ prey at night more efficiently on crustaceans in the presence of luminescent dinoflagellates.^15, 16, 18–21^

The bioluminescence of dinoflagellates is induced by a velocity gradient of fluids and can be utilized as an optical indicator for hydrodynamic and shear stresses.^14, 22^ Experiments showed that the intensity of the emitted light is a function of the shear stress level and cell concentration.^23^ Due to their size (approx. 250–400 µm) and geometry (spherical shape) the immobile dinoflagellates *Pyrosystis noctiluca* used in our experiments are comparable to other single cell systems in suspension. In this study, dinoflagellates were applied as reporters to identify influences of shear forces on single cells when exposed in a fast rotating clinostat and a RPM.

## Results

### Control experiments

**Fig. 2:**
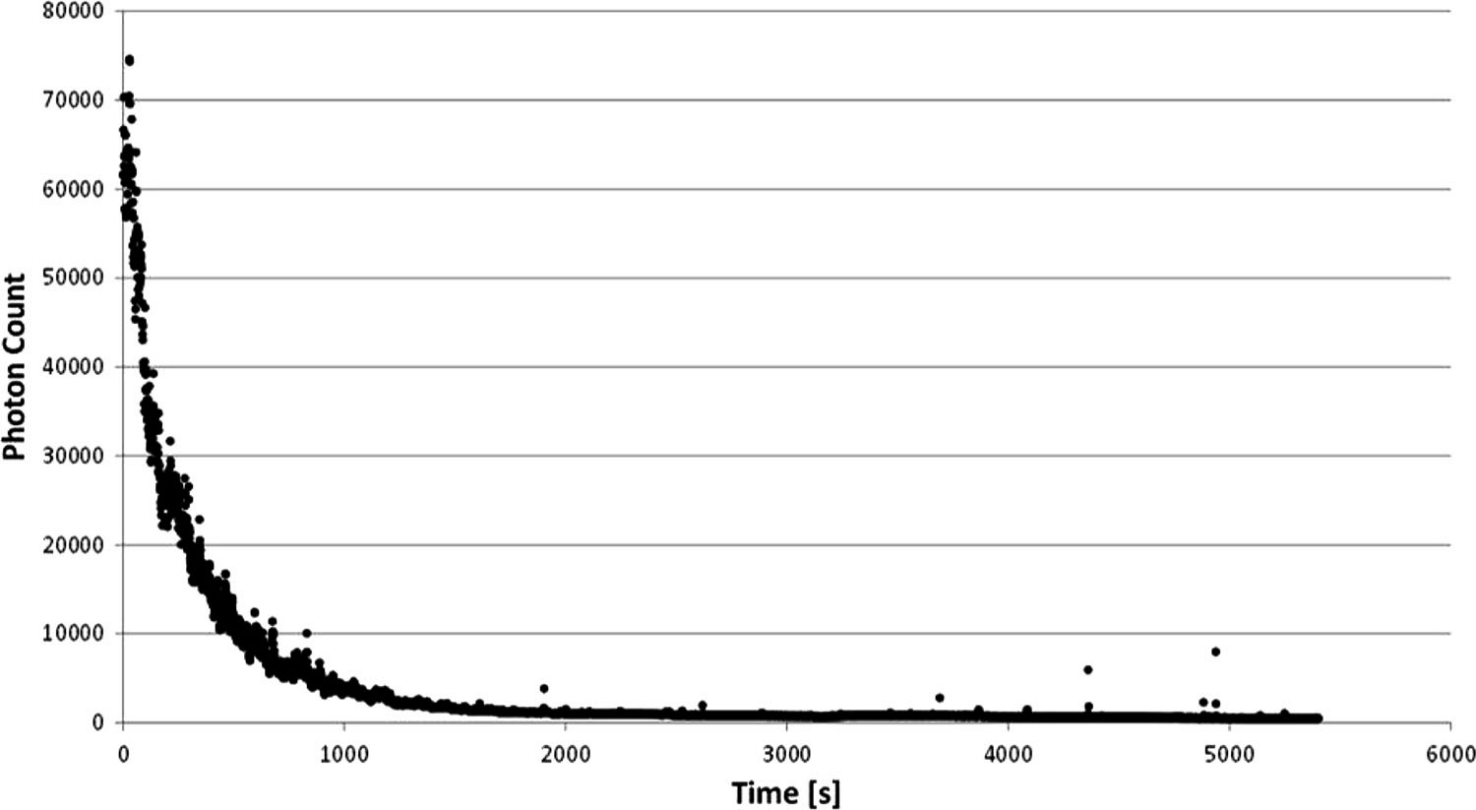
Representative relaxation curve of emitted bioluminescence of Pyrocystis noctiluca after transfer of the dinoflagellates into the sample cuvette. After 1 h, the emitted bioluminescence has stabilized and clinostat and random positioning experiments were started. *x*-axis: time in seconds, *y*-axis: number of photon counts

Control experiments were performed using dinoflagellates freshly filled into the glass cuvette. Immediately after zero head space-filling of the cuvette, the photomultiplier (PMT) recorded the photons of the bioluminescence produced by the dinoflagellates. A decreasing signal was monitored. In Fig. 2a typical relaxation curve is shown. After 1 h, the signal has stabilized. After this adaptation time the experiments were started.

### Clinorotation at 60 rpm

**Fig. 3:**
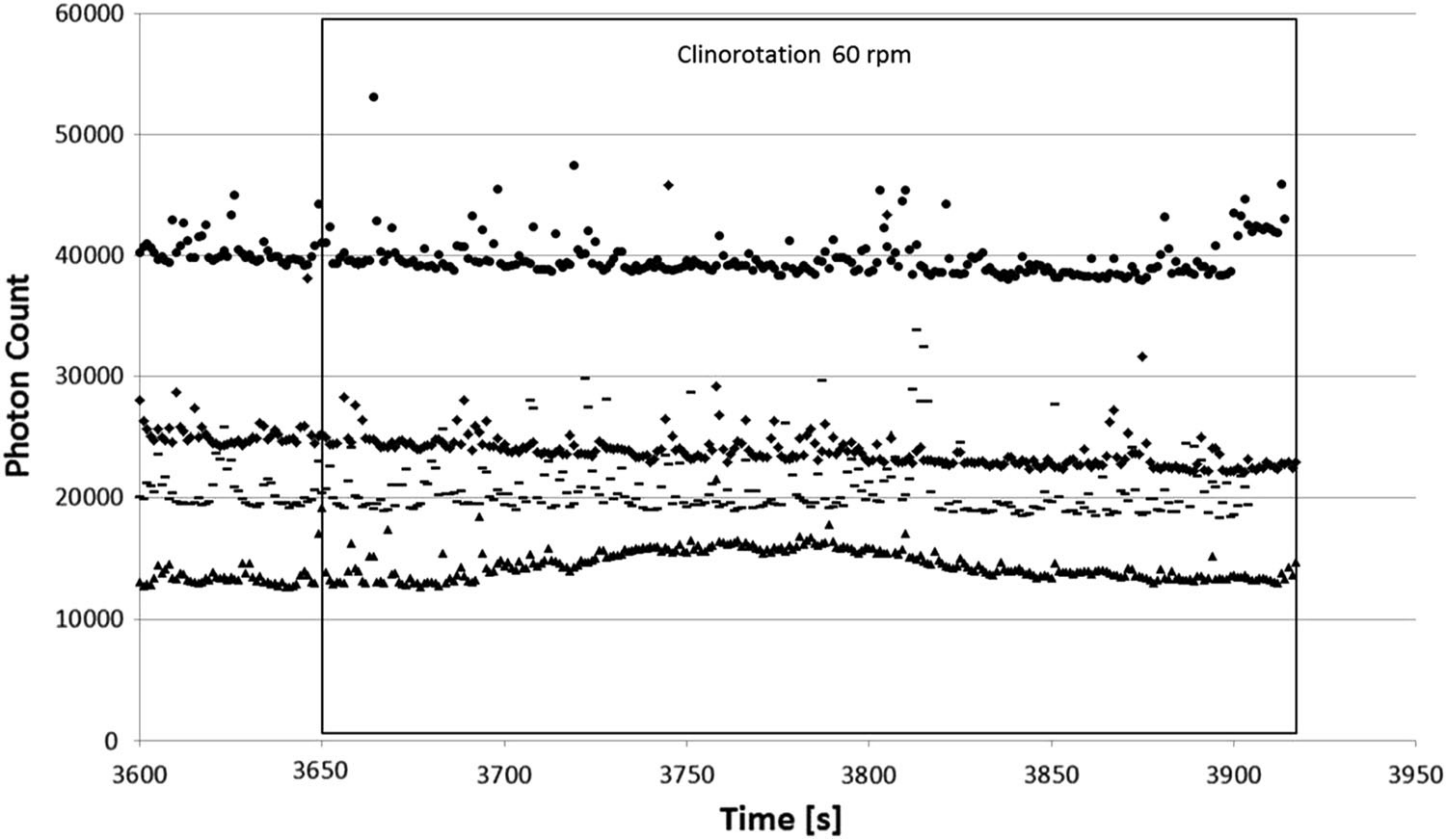
Bioluminescence curves showing four experiments in clinorotation mode (60 rpm, constant rotation, one axis) started after 3600 s of relaxation time (Fig. 2). The number of cells determines the baseline of photon counts. *x*-axis: time in seconds, *y*-axis: number of photon counts

After the relaxation time of 3600 s and thus stable level of emitted photons, the clinostat was started (3650 s) and the dinoflagellates were constantly rotated around one axis at 60 rpm. Four experimental runs (Fig. 3) reveal that clinorotation has no severe impact compared to the stable level of photons. The differences in the baseline of photon counts are due to different cell concentrations, as seen in the four examples in Fig. 3.

### Real random mode

**Fig. 4:**
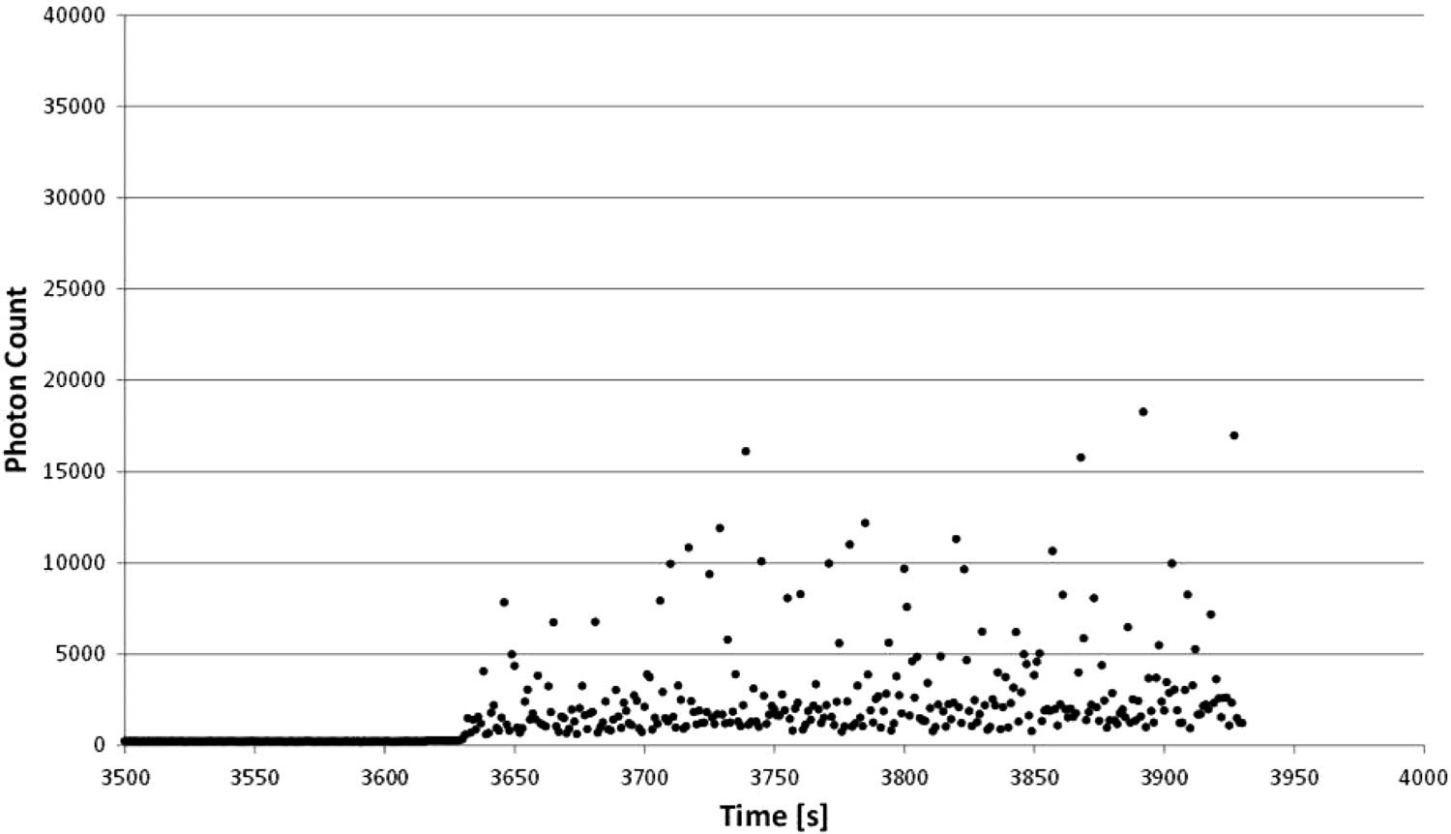
Complete counting of photons during an RPM experiment with random speed but unidirectional, that means without changing the direction of rotation. The RPM was started after 3625 s

**Fig. 5:**
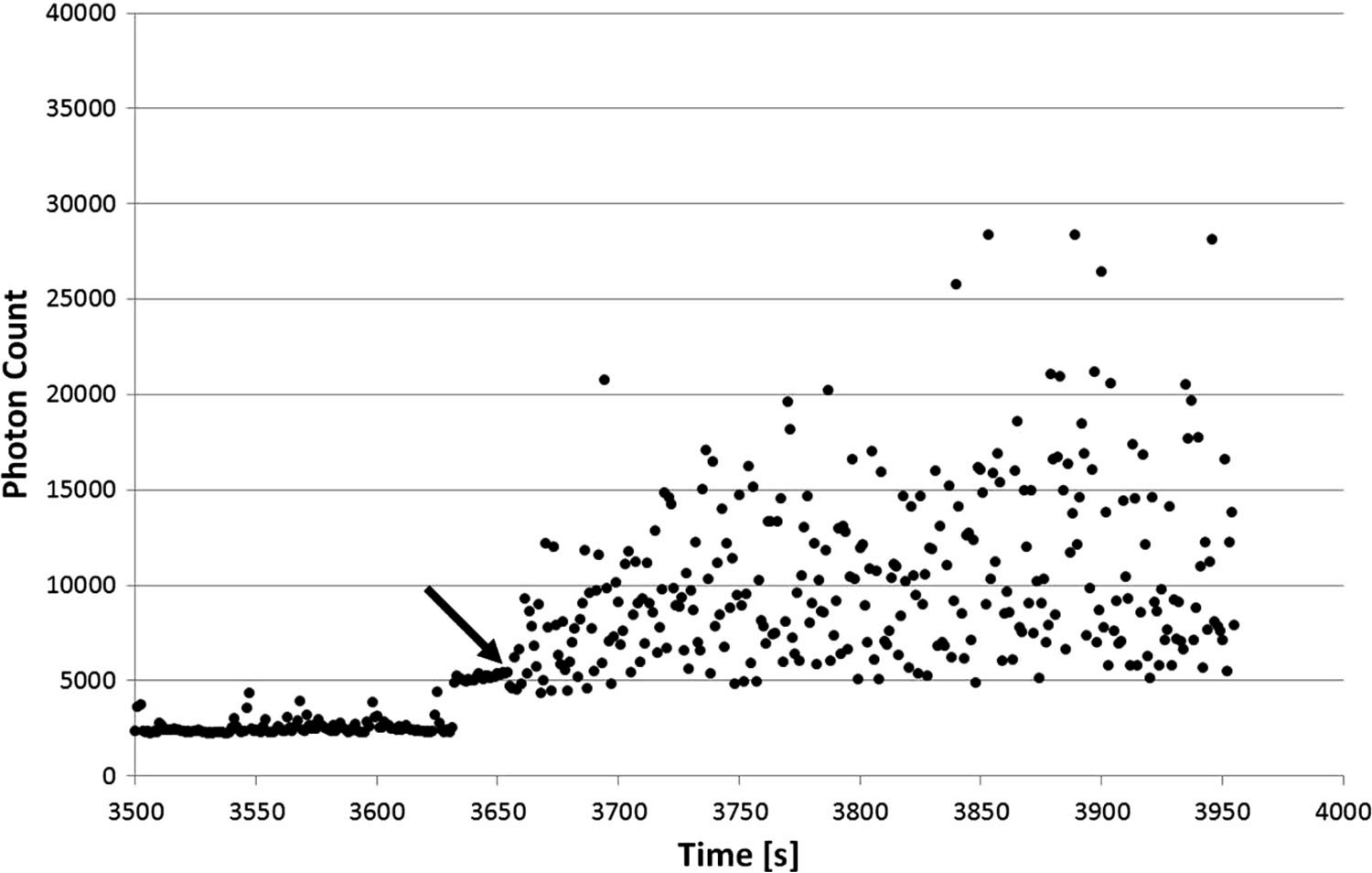
Photon counts of the bioluminescence signal under the RPM mode (random speed and random direction). The RPM mode started at 3635 s. *Arrow* indicates the first change in direction of rotation. The *x*-axis shows time in seconds, the *y*-axis shows photon counts

Using the identical experiment procedure and time line, we exposed dinoflagellates to random positioning by rotating those around two axes. Two kinds of random modes were applied. In a first set, the two rotating frames were operated at random speed without changing their rotation direction (unidirectional) (Fig. 4). In a second set, additionally to random speed the direction of rotation was changed at random (Fig. 5). Turning the RPM on, in both random modes, induces an increase in bioluminescence, which persists during the experimental time. It is obvious that the combination random speed and random direction has a stronger impact than just changing the speed at random. Furthermore, the first change of direction (Fig. 5, arrow) has a severe impact on the amount of emitted photons.

**Fig. 6:**
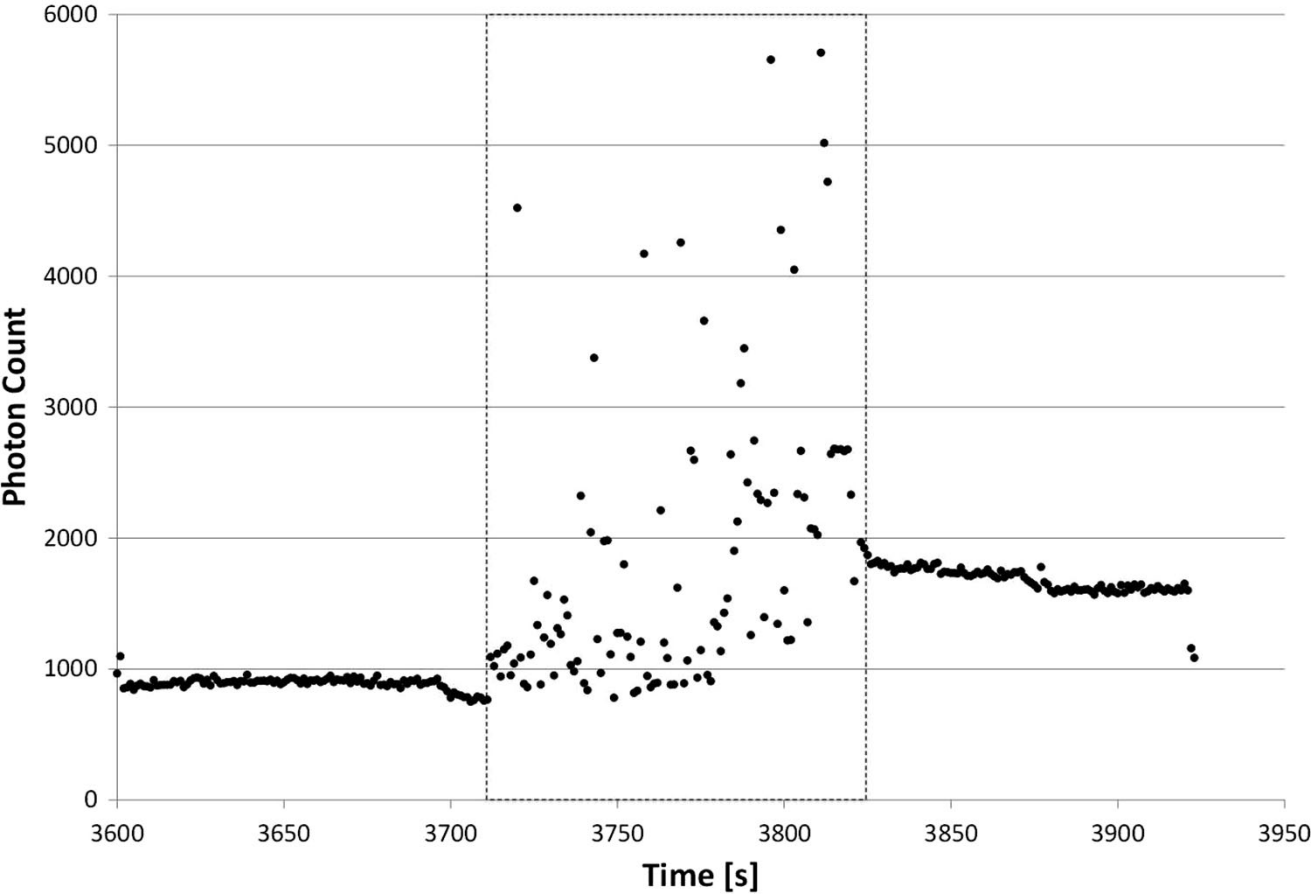
Direct impact of operation mode on photon emission. From 3600 s to 3713 s clinorotation (60 rpm) was applied, followed by random positioning with random speed and random direction (*dashed box*). Thereafter clinorotation was started again at 60 rpm

Clear evidence, that the operation mode has a direct impact on the amount of emitted photons, is given by repetitively switching between the modes (Fig. 6). The RPM platform allows us to expose one sample to both operation modes in the same run. Again, the operation was started after an adaptation of the dinoflagellates for 3650 s (compare Fig. 2). Starting first with the clinostat mode (unidirectional and turning with constant speed of 60 rpm) has no severe impact on the photon emission. A clear increase in emission is obvious immediately after addition of the second rotation axis, operated at random speed but constant direction. Switching again to the clinorotation mode immediately decreases photon emission. An increase in bioluminescence is even more pronounced if the RPM is operated not only at random speed but also random direction (data not shown). Again, we started the experiment with clinorotation (2D), followed by the random speed and random direction and finally again clinorotation. In this experiment run it is obvious that the intermediate exposure to the random mode also influences the level of emitted photon during the second clinorotation phase.

## Discussion

Before performing expensive and rarely available experiments in space, ground-based facilities provide an essential step for testing the gravity-related behavior of biological systems, and support the formulation of a hypothesis about results to be obtained in real microgravity. Gravity is permanent and constantly present on Earth. Correspondingly, ground-based microgravity simulators aim at compensating the effect of gravity, such as sedimentation. Rotation is one principle to counteract sedimentation in a liquid system. Samples are either rotated around one axis (2D) or around two axes (3D). Depending on the operational mode, these platforms have been termed clinostat that means constant rotation in one axis or RPM in case that the velocity and additionally the direction is changed at random in two axes. The NASA rotating wall vessel is an additional platform used in gravitational biology, aiming to oppose gravity and to simulate space conditions. Herein 3d-cell cultures are grown in a vessel of several diameters rotated at a speed, which avoids their sedimentation. Depending on the density of the cell culture and the density of the surrounding medium, the speed to be applied may vary greatly.^2, 24^ Due to the technical limitations an adaption with respect of the lager diameter of the vessel of the PMT device was not applicable. In order to evaluate this system with respect to potential shear forces further experiments need an configuration change of the photon collecting aperture to measure all emitted photons over the larger diameter.

Ground-based microgravity analog experiments have technical limitations. Samples are commonly exposed to the rotating platform of choice aiming to achieve microgravity analog conditions for them, then the device is stopped, samples are removed and further analyzed in a fixed or living status under 1 g conditions. To take this problem into account, online fixation and kinetic recording are important to improve the quality of simulation.^2^ New methods such as ‘‘omics’’ technologies demand large quantities of exposed cell material. Multi-generation experiments need optimal cultivation conditions and medium changes, which can hardly be achieved during rotation of a cell culture in suspension in a small volume. The small volume positioned along the center of rotation is a precondition to keep fluid flow disturbing forces due to rotation as small as possible, which are a source for unexpected reactions of the sample cells.^4, 25^ The radius which is used for the samples in 2D clinostats is in general rather small, in the range of millimeters resulting at a speed of 60 rpm in a constant maximal acceleration of 10^−3^ g (ref. 26). Users of the RPM claim that the advantage of RPM vs. 2D clinostats is the possibility to use larger sample volumes and thus gain more material for analysis.

As theoretical background of the RPM operation is proposed that over time the trajectory of the gravity vector points in all directions calculated by an appropriate algorithm, meaning after a longer time, e.g., 30 min, the mean gravity reaches theoretical zero.^7^ Due to our experiences, this is not predicable for the reactions of a fast gravity sensing organism.

Final validation of the quality of simulation is done by experiments in space.^2^

Considering simulation devices, it seems that after they have been published once, their use has been established, judged as reliable and the underlying physical principles drift in the background. The weightlessness environment for a system is characterized by a force-free condition. Thus, compensation of gravity can be only effective if the simulation approach does not induce new forces to cell systems, which will introduce unexpected signaling and physiological responses. Recent experimental set-ups allow online kinetics during exposure by coupling of the clinostat or a RPM with PMTs or online observation by combination with microscopy.^2, 27, 28^ Thereby, new results are added to the discussion about induced non-gravitational effects. They enable us to visualize the localization and behavior of cells, whether they are kept in stable positions or whether they are shifted and drifted during operation of the rotating device.

Such kind of studies revealed that 2D clinorotation forces cells/particles on circular paths whose diameter decrease with increasing acceleration. In contrast 3D clinorotation/RPM resulted in shifting fluid flows, bounce and moving out of the rotation center and changing accelerations of the rotated particles.^1^ Bumping and hitting of particles in the 2D clinostat was not observed, while in the RPM this phenomenon cannot be excluded. If this results in an increasing photon count is not known yet. Choosing an appropriate speed of rotation results in a similar distribution patterns, due to compensating of sedimentation as observed in real microgravity.^1^

Which are appropriate parameters of validation? Offset of a graviresponse must not be identical with a stimulus-free condition. Plants do no longer show gravitropic bending, independent of whether they are in real microgravity, under fast clinorotation or omnilaterally gravistimulated in a slow rotating clinostat. Though the graviresponse is no longer visible, earlier in the signal transduction chain, differences on the perception level can be predicted.

Dinoflagellates are a fast and sensitive reporter system for shear stress and hydrodynamic gradients acting as a deformation of the cell membrane resulting in a detectable bioluminescence light reaction visualized by means of photon counting probes.^9, 29, 30^ As a conclusion the emitted light of the dinoflagellates can be interpreted as direct measure of the intensity of perceived shear forces. Their response time was measured around 20 ms after mechanical impact on the cells and is described as one of the fastest reporter systems in nature.^9^ The sensitivity of dinoflagellates was tested in *P. lunula* by atomic force microscopy and results in a threshold of 7.2 ± 3.4 µN after a cell deformation of 2.1 ± 0.65 µm by a deforming area of 1.4% of the cell surface.^31^ This confirms the spherical-shaped dinoflagellates as ideal reporter in liquid media to visualize occurring side effects in the form of shear forces and velocity gradients in different operation modes of ground-based facilities, aiming to achieve microgravity analog conditions.

*P. noctiluca* shows a circadian rhythm, which results in the ability to flash and glow starting 1 h after the onset of darkness in the night. The ability of flashing and glowing is separated during the dark phase of the circadian rhythm. During the first 6 h mainly the flashing will occur induced by mechano-stimulation, while the last 6 h are dominated by glowing of the cells.^32^ This was considered in the preparation and time schedule of our experiments and we consequently performed all experiments within the first 6 h after the onset of darkness. So the primary reaction of shear forces resulted in flashing of the cells.

Our results indicate that the amount of mechanical stress is higher in the two axis RPM modes than during one axis clinorotation. This was shown in the higher photon count after starting the RPM mode after 1 h of relaxation time for the cells. A further indicator is the observed photon emission during the change of modes in the same run. The emitted photons immediately increased during random positioning and decreased after returning to the clinostat mode. This shows that higher residual forces are acting as shear forces on the cells during the RPM modes

The conclusions from our results imply that one axis clinorotation induced negligibly small side effects in the form of shear forces compared to the random modes with respect to velocity and direction using two axes. In contrast, both 3D random operation modes resulted in a high emission of photons by the dinoflagellates. These results should be considered by assessing further experiments on the RPM in the random velocity mode with and without random direction. It can be assumed that the observed shear forces impact on the signaling pathways or metabolic reactions interfering with most sensitive graviresponse reactions.^33^ A superimposition of intracellular signals cannot be excluded and should be kept in mind by planning and performing a ground-based experiment. As demonstrated in our experiments, a small sample cuvette will inhibit side effects (clinorotation) or even allow them (RPM) depending on the choice of operation mode.

Due to the fact that many groups using an RPM with cell culture flasks and thus large volumes, further experiments should be performed using a photon count system to obtain more results from the behavior of shear sensitive cell systems.^3^ Additionally, cell culture flasks have non-spherical shape and refer to a cuboids shape with the ability to generate large unpredictable turbulences inside the flask.

Ground-based facilities provide opportunity to prepare space experiments and learn about the sensitivity and behavior of the biological system of interest. However, operation modes should be carefully considered in order to avoid misinterpretation of results.

## Material and methods

Cell cultures of *Pyrocystis noctiluca*, obtained from the Culture Collection of Algae at the University of Cologne, were cultivated in T75 cell culture flasks in seawater with half strength F/2 media composition and without silicate solution in an illuminated incubator with a light:dark cycle of 12:12 h (refs 22, 34). The intensity of bioluminescence of *P. noctiluca* is coupled to a circadian rhythm. As a consequence the light dark cycle was set to 12 h of light from 8 p.m. to 8 a.m. so that the experiments could be performed during daytime. All experiments started after 9 a.m. in order to synchronize the cells to emit bioluminescence after stimulation by shear forces.^35, 36^

Small glass cuvettes (diameter 4 mm) were filled with a cell suspension of *P. noctiluca*. Special care was taken to avoid bubbles and to completely fill the chambers in order to prevent mechanical stress during rotation. The cuvettes were sealed with a rubber plug. To prevent a higher pressure inside of the glass cuvette a small stainless steel tube (0.4 mm diameter) was inserted inside the rubber plug to release overpressure, but keep the cuvette completely filled.

To quantify the bioluminescence of the treated dinoflagellates a digital PMT (Hamamatsu, Japan, H7155) was used as a photon detector. The counts produced by the internal logic of the PMT were processed with an Arduino Nano 3.0 (Reichelt Elektronik, Germany) and a counter program based on the *FreqCounter* library from Martin Nawrath from the Academy of Media Arts Cologne. With this library, the Arduino is able to count frequencies of up to 8 Mhz with a duty cycle of 50% on the digital port 5 (interface.khm.de/index.php/lab/interfaces-advanced/Arduino-frequency-couter-library/). The PMT logic sends one impulse (TTL, transistor-transistor logic) for every four counted photons. The used count divider based on a 74HC193 (Reichelt Elektronik, Germany) divides the signal by four and works as an impedance converter (Fig. 1d). With this divider chain every 16th photon is counted. An SD card stores the received counts send from the Arduino over the serial peripheral interface (SPI) to a text file with time (seconds) in comma-separated values (csv). The Arduino was programmed with a measure-time pitch of 1 s. The power supply is based on a lithium polymer accumulator with a step-up converter (Pollin Elektronik, Germany) to produce the 5 volt for the Arduino, the SD card and the PMT. To charge the lithium polymer accumulator of this portable device a universal-serial bus (USB) loader with mini USB connector (Pollin Elektronik, Germany) was integrated. Every subsystem was mounted on a laboratory-circuit board.

The PMT and the sample cuvette were installed in a light-tight box on the RPM. The center of the spin axis from the inner RPM frame and the rotational axis of the tube shaped cuvette were matched. Also the geometrical center of the glass cuvette was matched to the geometrical center of the inner RPM frame. The PMT window with the effective area was placed in direct contact to the glass cuvette. In this accommodation all photons reaching the PMT window will be counted.

### Modes of operations

The RPM was operated in three different modes:

Clinostat mode: 60 rpm, inner frame perpendicular to the gravity vector and rotating constantly. We selected the inner frame as it produces less vibration than the outer one (data not shown).

RPM: rotation of two axes mounted in a gimbal manner with random speed (with a highest speed of 60°/s, which is equivalent to 10 rpm) without random direction.

RPM: random speed (with a highest speed of 60°/s, which is equivalent to 10 rpm) with random direction.

### Software

For controlling the RPM inside the incubator the Windows program *RPMDesktop Controller* from Dutch Space was used in version 1.4.1.
